# Analysis of the Perception of Nutrigenetics in Conventional Nutritional Practice: A Survey-Based Study Applied to Dietitians from Mexico

**DOI:** 10.3390/nu17172776

**Published:** 2025-08-27

**Authors:** Diana Alejandra Vela-Vásquez, Ivan Delgado-Enciso, Janet Diaz-Martinez, Ana María Sifuentes-Rincón

**Affiliations:** 1 Laboratorio de Biotecnología Animal, Centro de Biotecnología Genómica, Instituto Politécnico Nacional, Reynosa 88710, Tamaulipas, Mexico; dvelav1600@egresado.ipn.mx; 2 Facultad de Medicina, Universidad de Colima, Colima 28040, Colima, Mexico; ivan_delgado_enciso@ucol.mx; 3 Department of Dietetics and Nutrition, Research Center in Minority Institutions, Florida International University (FIU-RCMI), Miami, FL 33199, USA; jdimarti@fiu.edu

**Keywords:** nutrigenomics, nutrigenetics, clinical practice, personalized nutrition

## Abstract

Background/Objectives: Nutrigenetics has emerged as a promising tool to advance personalized nutrition strategies. This study aimed to analyze the scope and perception of Mexican dietitians regarding nutritional genomics with an emphasis on nutrigenetics’ use in clinical practice. Methods: A survey was conducted online among dietitians in Mexico to assess their educational background, awareness of nutrigenetic testing, use in practice, and interest in further training through 33 questions. Results: One hundred and thirty participants from states across six Mexican regions completed the survey, and most of respondents had a bachelor’s degree. The analysis showed that while most respondents were familiar with the concepts of nutrigenomics and nutrigenetics, 92.3% did not incorporate genetic testing into their practice; the main barriers of their use were misinformation, limited access to reliable resources, and ethical concerns surrounding genetic testing. Although 86.2% expressed interest in learning about nutrigenetics, only 31.5% were willing to invest in further training. Social media and non-academic sources were important sources of information, raising concerns about their inaccurate content and highlighting their importance in completing the curricula. Patients’ demand for genetic testing is limited and directed by disease prevention interests. Conclusions: Nutrigenetics is currently an area with limited practical application among Mexican dietitians; however, it is perceived as a valuable tool for future daily practice. The gap between perception and application underscores the need to integrate nutrigenetics into undergraduate curricula and to provide accessible, evidence-based professional development; these are essential to promote the ethical and effective use of nutrigenetics and support the transition toward personalized nutrition.

## 1. Introduction

In recent years, advances in biological and genetic sciences have allowed us to acquire a better understanding of the relationship between food and genes, leading to the emergence of nutritional genomics. This branch of science studies how food and its components interact with the human genome, influencing genetic expression and therefore health and well-being [1]. Through two different approaches, i.e., nutrigenomics and nutrigenetics, this science has opened a new era in personalized nutrition where dietary recommendations are not only based on demographic or clinical factors but also on individual genetic information, potentially improving overall health outcomes [2,3]. While nutrigenomics focuses on the effects of nutrients on gene expression, nutrigenetics aims to determine how genetic variations impact an individual’s response to nutrients [4].

The degree of acceptance and application of nutrigenetics by dietitians still varies considerably by region and available resources [5,6]. To date, some countries, such as the US, Canada, Spain, and Australia, have commercial nutrigenomic services. Unfortunately, many dietitians around the world are still far from having the appropriate knowledge and skills to effectively integrate these new tools into their professional practice [5,6,7]. Therefore, there is a common worldwide interest in developing strategies to support the integration of these new approaches into daily nutritional practice.

In Mexico, both private and public educational institutions have been integrating nutritional genomics into their curricular plan’s courses, diploma, and postgraduate programs (e.g., INMEGEN, EP de México, Instituto de Nutrigenómica, Tecnológico de Monterrey, UANL, UAT). These programs, among others, aim to provide health professionals specializing in nutrigenetics with a solid foundation in nutrition, basic sciences, and genetics, encouraging them to apply this knowledge in their clinical practice [8]. However, as has been reported for other countries, Mexican dietitians still demonstrate skepticism about the application of nutrigenetics, mainly due to a lack of regulation of websites offering nutrigenomic testing and dietary strategies based on it [9]. These communication channels have caused misinformation and have led to negative perceptions of these tools and to their categorization as “pseudoscience” [9].

This study aims to analyze the scope and perception of dietitians from Mexico regarding using nutrigenetics in their clinical practice. Via descriptive research, we seek to gather information about the use of commercial nutrigenomic testing in nutritional practice, the current demand from patients to use nutrigenetic testing, and the resources they use to apply and interpret the results of testing in their management of patients.

## 2. Materials and Methods

### 2.1. Study Participants and Recruitment

A non-probability for convenience sampling method was used. Dietitians from Mexico were invited to participate in an online survey via Google Forms. An exclusion criterion was having studied nutrition outside of Mexico. Informed consent was obtained through the Google Form, which confirmed who was responsible for creating the survey, its purpose, and the anonymity of the responses. The study and the questionnaire used were approved by the Research Ethics Committee of Instituto Estatal de Cancerología de Colima (CEICANCL2024-PERCEPS-11; 12 April 2024). The survey was distributed through various associations, social media groups, and networks of graduates and practicing dietitians to examine their knowledge of, attitude towards, and perception of nutrigenetics education and its use in their nutritional clinical practice.

Participants were graduates with a bachelor’s degree in nutrition and did not receive any compensation for their participation in the survey.

### 2.2. Experimental Procedure

The survey included thirty-three questions designed to assess respondents’ knowledge of nutritional genomics, the application of nutrigenetic testing, and their availability and interpretation, as well as to determine whether they received training on this topic during university or where they acquired this knowledge or training Supplementary Materials (Supplementary S1; Questionnaire). Additional questions evaluated both their desire to learn more about nutrigenetics and ethical considerations.

The survey included open-ended (for personal data such as age, university, and year of graduation), as well as closed questions with single or multiple-choice answers depending on the nature of each question. The survey was developed by a dietitian with a PhD in biotechnology and reviewed by colleagues in the field. A qualitative pilot test involving five dietitians with training in nutritional genomics reviewed the questionnaire as an initial validation test; the questions were edited to ensure that they were comprehensible, concise, and addressed the objective of the study [10]. Subsequently, a quantitative pilot test was carried out with the questionnaire, with 40 dietitians to assess internal consistency and correlation with the total item. Responses from the pilot tests were excluded from the final data analysis in this study.

For consistency in the item–total correlation, a minimum value of 0.3 was considered acceptable. All questions showed a reasonable standard deviation, avoiding highly homogeneous or constant values. One question was eliminated due to low variability and three due to low correlation.

To guarantee reliability, internal consistency was measured using Cronbach’s alpha, grouping the questions into the following dimensions: knowledge and training, clinical practices and attitudes, and technical knowledge and genetic perception. The resulting Cronbach’s alpha coefficients were acceptable. Even the resulting coefficient values were acceptable (0.650, 0.750, and 0.706, respectively). However, it is important to remark that questions 8 and 9 (“How did you learn about Nutrigenetics?” and “During your degree, did you take Nutrigenetics or a related course?”) showed a corrected item–total correlation of less than 0.3 and tended to reduce the Cronbach’s alpha value for the knowledge and training dimension. Despite this, we decided to retain these items in the questionnaire as they address specific and fundamental aspects not captured by other items, i.e., valuable information about knowledge sources and academic training in nutritional genomics. To avoid bias in inferential analyses, these questions were excluded from statistical analyses but were kept in the general description to maintain the integrity and comprehensiveness of the construct assessed.

The survey was created in Google Forms for online responses and distributed exclusively to graduated dietitians through social networks and direct contact. It was continuously shared to maintain visibility, accounting for a total of 84 days of availability throughout the year (03/2024 to 03/2025). To preserve anonymity, respondents’ names were not requested. However, due to Google Forms’ design, email addresses were collected to ensure a single response per participant. Nonetheless, full confidentiality of responses and publications was guaranteed, and access to the database was restricted to a single researcher responsible for data management and analysis, ensuring the privacy and security of participants’ information.

### 2.3. Statistical Analysis

A descriptive statistical analysis of respondents’ answers was conducted. The results were reported using counts and percentages, without thematic coding or theme extraction, enabling the identification of response trends within the sample analyzed. The item-total correlation for each question, and Cronbach’s alpha coefficients for each set of questions grouped by the dimensions examined, were calculated.

To explore associations between demographic, knowledge, and perceptions variables, Fisher’s exact test was performed. Additionally, odds ratios (OR) with their 95% confidence intervals were obtained through crosstabs and Fisher’s exact tests to evaluate the strength of associations. For this data analysis, the SPSS Statistics version 20 software program was used (IBM Corp., Armonk, NY, USA). A *p* < 0.05 was considered statistically significant.

## 3. Results and Discussion

### 3.1. Demographics

**Figure 1:**
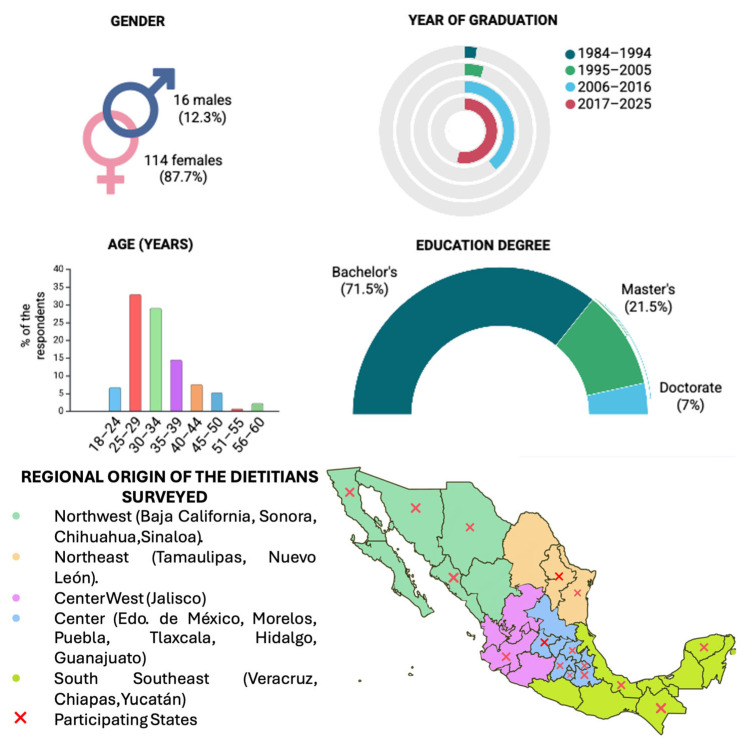
Demographics of the dietitians who participated in the survey.

One hundred and thirty dietitians responded to the survey. The participants were mainly women (87.7%), with an average age of 33.01 years, holding bachelor’s degrees and having graduated between 1984 and 2025. The demographics of the sample are shown in Figure 1. The percentage of nutritionists by state and their academic degree is shown in detail in the Supplementary Materials (Supplementary S2: State of origin and academic degree of the nutritionists surveyed).

The questionnaire was shared through different electronic platforms reaching professionals across the country. Professionals from Reynosa, Tamaulipas, accounted for 46.2% of the respondents (130). Overall, 62.3% were from the northeast (Tamaulipas, Nuevo León), 23.1% from the center (including Hidalgo, Estado de México, Morelos, Tlaxcala, Puebla, Guanajuato), 4.6% from the south and southeast (Veracruz, Chiapas, Yucatán), 5.4% from the northwest (Chihuahua, Sonora, Sinaloa, Baja California), and 4.6% from the rest of Mexico (Jalisco) (Figure 1). According to the data from the Mexican Secretariat of Economy for the third quarter of 2024 [11], this geographic concentration represented 5.5% of the total active dietitians (2359 dietitians) across ten participating states, ranging from 0.2 to 66.7% in the participating states; thus, their responses offer valuable insights into regional trends and the current state of awareness and interest in nutrigenetics.

In Mexico, the reported gender distribution for the nutrition profession indicates that 63.4% are women [11]. In this survey, a predominance of female respondents was observed (87.7%). The result is consistent with the feminization of the field.

Compared to other studies addressing the topic of nutrigenetics, our sample size is moderate. Previous research has included sample sizes ranging from 12 registered dietitians to 127 students (73 of whom were from Nuevo León, Mexico) [7], 155 dietitians from Grece [12], and up to 1844 dietitians from the US, Australia, and the UK [6]. While our survey does not match the scale of large international studies, it exceeds the sample size of some survey-based studies and remains informative; furthermore, the number of respondents (*n* = 130) highlights both the relevance of the topic and the level of engagement among Mexican dietitians. The survey was open access and remained available for nearly four months over two different years (2024 and 2025). This response rate reflects a meaningful degree of interest despite the voluntary nature of participation and the absence of incentives. Additionally, the sample includes professionals from various regions of the country, offering a reasonably representative perspective on the national context.

### 3.2. Level of Knowledge About Nutrigenetics Among Mexican Dietitians

Knowledge plays a crucial role in the adoption of new tools in professional practice; thus, the first point that we addressed was to determine the extent to which professionals are familiar with the concepts of nutrigenomics and nutrigenetics and with their tools. In this regard, most respondents reported being informed about nutrigenetics and nutrigenomics; however, only 6.9% of the 130 respondents had received practical training in the field, and only 6.2% applied it in research. To date and to our knowledge, there is no updated data on the involvement of dietitians in research activities in México. Available information from 2013 reveals that only 0.74% of researchers affiliated with the Sistema Nacional de Investigadoras e Investigadores conducted research in nutrition [13]. Although the low number is shocking, it is not unexpected, as prior reports indicate that few dietitians have research as a goal of their professional trajectory [14].

Interestingly, it was found that 54.6% of professionals declared that they had heard about nutrigenomics and nutrigenetics through academic institutions that were not necessarily their formation institution; 64.6% did not study it as a subject during their university career.

This is relevant given the updating of the programs. Companies have been commercializing nutrigenomics since the 1980s [15], and it has been introduced into the US and European curricula since 2010. It is important to mention that most of the academic offerings in nutritional genomic training are carried out in high-income countries, mainly the United States and the United Kingdom [16], which represents a disadvantage for students in Mexico and Latin America, since it has required time to integrate these offerings into most academic plans. Of the 84 respondents who did not study nutrigenetics during their degree, the majority were from the northeast (61.9%), followed by the center (23.8%), with lower representation from the south/southeast (7.1%), northwest (4.8%), and center west (2.4%). Additionally, 23.8% of them graduated before 2010, suggesting a possible relationship between the age of the curriculum and the lack of training in nutrigenetics. Among the 46 respondents who did take the course, 86.9% graduated, on average, around 2018, further supporting the idea that the integration of nutritional genomics into academic programs is a relatively recent development. The northwest, center west, and south/southeast are underrepresented in both groups and contain very few individuals who studied the subject, which may reflect a low integration of nutritional genomics into curricula or limited access to this type of training in those regions. The observed trends should be viewed considering the sample’s regional distribution, which may limit generalizability across the entire population of nutritionists.

Van Buren et al. [7] conducted a study comparing the interest in and knowledge of nutritional genomics topics among nutrition students from two populations, one in Texas, United States of America, and the second one in Nuevo Leon, Mexico, reporting that nutrition students from the USA and Mexico have a low level of knowledge in different omics topics such as metabolomics, proteomics, and nutrigenomics, but the interviewees recognized the role that omics will play in their future as dietitians, since in order to make personalized nutrition a reality, dietitians need to become fluent in different omics technologies. As Rosen et al. [17] state, “limited knowledge and confidence about genetics and nutritional genomics is likely to be a major barrier preventing the application of these concepts”.

Beyond academic exposure, the dissemination and perception of nutrigenetics have also been shaped by external sources. In our study, participants (130) reported acquiring information about these topics from scientific articles (36.9%), followed closely by the internet (32.3%), conferences (24.6%), and social media (23.1%). While the presence of peer-reviewed literature is promising, the high reliance on non-academic platforms raises concerns about the quality and accuracy of the information being consumed. In particular, platforms such as social media and commercial websites have contributed to the popularization of nutrigenetics without a clear clinical framework, especially in the context of weight-loss strategies. This trend may promote the use of genetic tests and personalized diets more as commercial tools than as interventions grounded in solid scientific evidence, which can underestimate the credibility of the field and delay its integration into evidence-based practice.

### 3.3. Application of Nutrigenetic Tests in Daily Practice

The second topic we addressed was whether nutrigenetic tests are used in daily practice. For 92.3% of the dietitians surveyed, nutrigenetic testing is not a common tool in their daily practice; most of them declared a lack of knowledge about the concept, its scope, and the importance of using these tools. Additionally, 50.8% were unaware of the existence of nutrigenetic tests on the market. Interesting, most of these 66 respondents were from the northeast (72.7% respondents), followed by 18.2% from the center and 3% each from the northwest, south/southeast, and center west, with an average age of 32 years, and most graduated around 2017. In contrast, those who were aware of the existence of the tests (49.2%, 64 respondents) were also mainly from the northeast (51.6%), followed by 28.1% from the center, 7.8% from the northwest, and 6.3% from the south/southeast and center west. Their average age was slightly higher, at 34 years, with an average graduation year of 2017. This result suggests that graduation year is not a determining factor for the use of nutrigenetic testing. However, access to continuing education (i.e., holding a master’s or doctorate degree) has a significant influence on awareness and familiarity with these tools (*p* = 0.003). A postgraduate degree increases 3.4 (OR 3.4, 95% CI 1.511–7.746) and 5.3 times (OR 5.3, 95% CI 1.787–16.139) the probability of knowing about nutrigenetic testing, and the use and interpretation of nutrigenetic testing results, respectively. Supporting this, among the 64 professionals who were aware of the use of nutrigenetic tests, 70.3% had pursued postgraduate studies, and 40.9% had a bachelor’s degree. On the other hand, among those unaware of the tests, only 29.7% had pursued postgraduate studies, and the rest (59.1%) only held a bachelor’s degree; however, due to the higher participation in certain regions, these results may reflect regional perspectives more strongly than the national average.

Interestingly, while most professionals studying a nutrigenetics subject during their university studies were from the northeast region, this same region also accounted for the highest number of professionals who were unaware of the existence of these tests. This apparent contradiction may be due to several factors: they may have taken the course with limited theoretical content, or the concepts may not be integrated into current professional settings. Future studies are recommended to explore in depth the different academic aspects and contextual barriers that could be influencing the integration of nutrigenetics into clinical nutrition practice.

This scenario emphasizes the need not only to include nutrigenetics in academic curricula but also to strengthen practical training and promote ongoing professional education; this has also been emphasized by Kalafati in Greece [12], reinforcing the urgency of promoting the curriculum’s updating to best meet current and future challenges and opportunities regarding nutritional genomics.

The results reveal a significant knowledge gap: more than half of those surveyed did not understand the relationship between the genome and eating habits (53.8%) or between the genome and body composition (42.3%). However, nearly three-quarters of them still considered nutrigenetic testing applicable to their professional practice. These results reinforce the idea that the current use of nutrigenetic testing is driven more by lack of information and social media marketing campaigns than by scientific understanding.

Nowadays, in the commercial landscape, nutrigenetic tests are often promoted with promises of personalized diets, an opportunity to end the one-diet-fits all approach, and improved health outcomes, particularly regarding weight loss and body composition, even when scientific evidence supporting such claims is still evolving [5,18]. Therefore, although many dietitians may not fully understand the underlying science, they may perceive the tests as useful tools simply because they are widely advertised as such and demanded by clients. On the other hand, the interest in learning about nutrigenetics is high. People are most frequently interested in understanding how genetic variants affect eating habits and body composition. Most professionals (86.2% of the respondents) are likely to pursue this knowledge (taking a nutrigenetics course). Integrating nutritional genomics into undergraduate nutrition programs appears to be the most effective strategy to ensure a solid scientific foundation and responsible application of these tools in Mexico’s clinical practice. This strategy should be led by professionals with solid technical and theoretical backgrounds in nutritional genomics. By introducing this content early and from reliable academic sources, future professionals should be able to evaluate genetic testing, differentiate pseudoscientific claims from evidence-based findings, and thus develop better personalized nutrition strategies.

### 3.4. Patient Interest in Nutrigenetic Testing

We also addressed whether patient interest in nutrigenetic testing could drive its use in daily practice. The data indicates that patients do not currently request these tests to support their nutritional management. However, 12.3% of respondents reported that patient interest in testing has different motivations (Table 1). This was especially true in the northeast and central areas. Although the questionnaire did not include questions addressing how patients accessed information about nutrigenetic testing or its perceived benefits, we can assume that this information may have reached patients through digital media, social networks, or direct marketing, where genetic testing is frequently promoted for disease prevention. In this sense, it cannot be ruled out that the marketing discourse used by companies offering these services is influencing the perceptions of both patients and some dietitians, since 95 dietitians surveyed consider nutrigenetic tests applicable in consultation but do not know how the genome affects eating habits or body composition. The lack of sufficient knowledge and interpretative skills could result in the delivery of incomplete or potentially inaccurate information to patients, which may compromise the quality of care. Without proper training, the use of these tests could unintentionally lead to practices that, while well-intentioned, may be misleading or not fully aligned with scientific evidence.

Interest from patients in the application of nutrigenetic testing raises questions about the scope of their use in diagnosis or treatment by dietitians. In this respect, most of the dietitians surveyed (86.2%) reported that they are not qualified to provide additional genetic counseling to their patients if the results of nutrigenetic tests reveal further genetic risks. Fortunately, 90.8% of respondents are willing to refer their patients to a specialist or genetic counselor if necessary, without distinctions in academic level with 89.2% (83 dietitians) of those with a bachelor’s degree and 94.6% (35 dietitians) of those with a postgraduate degree (*p* = 0.507) indicating that they would be willing to do so) or between the regions (*p* = 0.409). This is an important step, as health professionals frequently face challenges when deciding whether to refer patients to specialists or genetic counselors for nutrigenetic testing. While many patients appreciate having a health professional devoted to interpreting their nutrigenomic test results, some health professionals experience tension between supporting their patients and questioning the clinical validity and utility of these tests. This has been previously discussed, and our study confirms that although there is a willingness to provide referrals, concerns about the reliability of the tests and the accuracy of information provided by marketing companies often accompany these decisions [16,19].

Most importantly, genetic testing should not be requested without appropriate guidance or a clear understanding of its implications, further highlighting the need for both basic and specialized genetic training within the academic curricula of dietitians. Therefore, the development of clear protocols to guide patient referrals and promote interdisciplinary management in the use of nutrigenetic testing is essential. These protocols should establish ethical, clinical, and professional competency criteria, facilitating collaboration among dietitians, geneticists, and other healthcare professionals to ensure accurate interpretation and personalized, evidence-based interventions.

### 3.5. Ethical Concerns and Nutrigenetic Testing Regulations

Although the demand for nutrigenetic tests is increasing globally, especially through “direct-to-consumer” (DTC) services—genetic tests marketed directly to consumers without healthcare professional involvement—these services are associated with various legal violations and potential consumer fraud [20].

One of the main issues with DTC tests is that although they are sold globally, they are not necessarily validated for all racial or ethnic groups. This critical information must reach all professionals who apply these tests, as they could be deceiving their patients, especially in Mexico and Latin America, where most of these tests are not population-validated; thus, there is no certainty about the effect of polymorphisms included in each testing. On this basis, we found that most dietitians surveyed (85.4% 111) considered it unethical to promote and apply DTCs, as well as nutrigenetic tests, when they have not been validated for the Mexican population [21]. Nevertheless, while informative, these perceptions may not fully represent the views of dietitians nationwide due to the sample’s regional distribution.

Although critical ethical aspects about legal regulation and supervision of laboratories accredited to perform medical genetic testing, this topic is not addressed in the survey questionnaire. It would be interesting to determine, through future studies, if dietitians are aware of the regulations and accreditation standards necessary to judge the reliability and clinical validity of genetic testing services, as this knowledge could directly influence their confidence in integrating such results into nutritional practice.

Finally, all the results and discussion of our study have an important limitation: the use of non-probability sampling does not ensure the representativeness of the entire population of nutritionists in Mexico. Relying on self-selection, dissemination through social media may have primarily attracted professionals with a greater interest in or familiarity with the topic. Additionally, since the survey was mainly distributed via social media and academic networks, the participation of professionals with limited access to these platforms may have been restricted. Over-representation of participants from northeastern states limits the study; thus, results should be interpreted with caution, as they may not fully reflect the perspectives of nutritionists from other regions. Future research should employ more representative sampling strategies to improve generalizability.

## 4. Conclusions

In Mexico, nutritional genomics is an emerging science that continues to possess limited practical applications among Mexican dietitians who perceive it as a future valuable tool for daily practice. The data shows that while there is growing interest in nutrigenetics as the basis of personalized nutrition, academic issues are important factors limiting its use; therefore, it is essential to develop strategies to incorporate this discipline into undergraduate nutrition curricula within all the country’s institutions and to increase postgraduate offerings in nutritional genomics. In addition, in order to promote consistent and sustainable application of these tools, it is imperative to initiate—with national policies focused on guaranteeing standardized competencies ensuring equitable and ethical access—the application of nutrigenetics in conventional practice.

## Figures and Tables

**Table 1:** Reasons why patients have become interested in nutrigenetic testing.

Reason	*n* (%)
Curiosity	9 (6.9%)
Desire to change or modify their diet based on the result	12 (9.2%)
Marketing	3 (2.3%)
Disease prevention	16 (12.3%)
Not interested	90 (69.2%)

## Data Availability

The data presented in this study are available on request from the corresponding author due to questionnaires may contain confidential information about survey participants.

## Supplementary Materials

The following supporting information can be downloaded at https://www.mdpi.com/article/10.3390/nu17172776/s1. Supplementary S1: Questionnaire, Supplementary S2: State of origin and academic degree of the dietitians surveyed.

## Abbreviations

The following abbreviations are used in this manuscript:

INMEGEN	Instituto Nacional de Medicina Genómica
UANL	Universidad Autónoma de Nuevo León
UAT	Universidad Autónoma de Tamaulipas
US	United States
UK	United Kingdom
DTC	Direct-to-Consumer
