# Supplementary material for: Analysis of the Perception of Nutrigenetics in Conventional Nutritional Practice: A Survey-Based Study Applied to Dietitians from Mexico
Source: Nutrients. 2025 Aug 27;17(17):2776. doi: 10.3390/nu17172776 (PMC12430391; doi:10.3390/nu17172776)
Supplement: Supplementary file 1 [file nutrients-17-02776-s001.zip › nutrients-3794986-supplementary.pdf]

Supplementary Materials

Supplementary S1: Questionnaire.

Hello, the Laboratorio de Biotecnología Animal del Centro de Biotecnología Genómica invites you to participate by completing the following questionnaire. This questionnaire aims to document nutritionists' knowledge of the application of nutrigenetic concepts in their daily practice. Your responses are confidential and will be used solely for research purposes.

1. Gender (single choice)

-Male

-Female

2. Age (open question)

3. Highest level of education (single choice)

-Bachelor's

-Master's

-Doctorate

-Specialization

4. Year in which you graduated from your bachelor's degree (open question)

5. Name of the University from which you graduated (open question)

6. Have you heard of Nutrigenomics / Nutrigenetics? (single choice)

-Yes

-No

We are currently in the era of molecular nutrition, where gene-nutrient interactions are studied. There are two important areas within this field: Nutrigenomics, which studies the influence of nutrients on gene expression, and Nutrigenetics, which studies the influence of genetic variations on the organism's response to nutrients (Gómez Ayala, 2007).

7. What do you consider to be your level of knowledge on the subject? (multiple choice)

-I'm familiar with the concept

-I've trained in the area through theoretical courses

-I've trained in the area through theoretical and practical courses

-I apply it in research

-I'm unfamiliar with it

8. How did you learn about Nutrigenetics? (multiple choice)

-Social media

-Academic institutions

-Conferences

-Internet

-Books

-Scientific articles

-I didn't know about it

9. During your degree, did you take Nutrigenetics or a related course? (single choice)

-Yes

-No

10. Do you think social media is a good way to educate patients and nutrition professionals about nutrigenetic testing and its implications? (single choice)

-Yes

-No

11. Do you provide nutritional counseling? (single choice)

-Yes

-No

12. Public or private practice? (single choice)

-public

-private

-I don't practice

13. Are you aware that nutrigenetic tests are already commercially available? (single choice)

-Yes

-No

14. Have you ever taken a nutrigenetic test? (single choice)

-Yes

-No

15. Do you consider nutrigenetic testing applicable in your practice? (single choice)

-Yes

-No

16. Do you consider nutrigenetic testing applicable in your practice? (single choice)

-Yes, I have performed the tests and requested informed consent.

-Yes, I have performed the tests but have not requested informed consent.

-I have not performed the tests.

17. How do you apply the results of the Nutrigenetic test in your consultation? (multiple choice)

-Used solely for informational purposes for the patient

-I develop your nutritional plan based on the results, focusing on mutations related to metabolic syndrome

-I develop your nutritional plan based on the results, focusing on mutations related to alterations in biochemical parameters

-I develop your nutritional plan based on the results, focusing on body recomposition strategies

-Used solely to provide a more complete patient history

-I do not order nutrigenetic testing during consultations

18. What do you think is the relationship between a patient's genome and their eating style (greater preference for a particular macronutrient)? (multiple choice)

-Some variations in the genome cause a higher consumption of X macronutrient.

-Some variations in the genome could cause a higher consumption of X macronutrient, but this has not yet been confirmed.

-Variations in the genome do not cause a higher consumption of any macronutrient.

-I'm unfamiliar with the topic.

19. What do you think is the relationship between a patient's genome and body composition? (multiple choice)

-The genome or variations in the genome do not influence body composition.

-It is possible to meet the patient's goals through genetic knowledge.

-The genome or variations in the genome influence body composition.

-The patient's genome influences waist/hip circumference.

-I'm unfamiliar with the topic.

20. Do you think nutrigenetic testing could help you analyze, diagnose, or treat a patient's biochemical and physical parameters? (single choice)

-Yes

-No

21. What is your main motivation for promoting or not promoting these tests? (multiple choice)

-Identifying mutations that are associated with alterations in biochemical parameters.

-Generating nutritional plans based on the patient's genetic information.

-Providing added value to the consultation.

-Increasing the price of the consultation.

-All of the above.

-I have no interest in promoting these tests.

22. What is the main reason your patients have shown interest in Nutrigenetic test? (multiple choice)

-Curiosity about the topic

-Desire to change or modify their diet based on the results

-Disease prevention

-Marketing

-They haven't shown interest

23. Do you think the test results have or will have a positive or negative impact on your patient? (single choice)

- Positively

- Negatively

24. Do you know how to interpret and apply the results of the nutrigenetic test? (single choice)

-Yes

-No

25. Are you sure your patients fully understand the nature and limitations of nutrigenetic test? (single choice)

-Yes

-No

26. Are you prepared to handle your patients' sensitive genetic information confidentially and securely? (single choice)

-Yes

-No

27. Are you committed to using the results of Nutrigenetic test responsibly and based on scientific evidence? (single choice)

-Yes

-No

28. Are you qualified to offer additional genetic counseling to your patients if nutrigenetic testing results reveal additional genetic risks? (single choice)

-Yes

-No

29. Are you willing to refer your patients to a genetic counselor or other healthcare professionals if their nutrigenetic testing results require specialized care? (single choice)

-Yes

-No

30. Would you be willing to take a training course in Nutrigenomics? (single choice)

-Yes

-No

31. What would you like to learn about nutrigenetics? (multiple choice)

-The effects that genetic variants can have on eating habits.

-The effects that genetic variants can have on body composition, for example, a higher percentage of body/abdominal fat, greater muscle mass, etc.

-The effects that genetic variants can have on eating habits and body composition.

-How nutrigenetic testing is performed (techniques)

-I have no interest in the field.

32. Are you committed to staying up-to-date on advances in nutrigenetics and regularly reviewing your ethical practices in this field? (single choice)

-Yes

-No

33. Do you consider it ethical to sell/promote a test that has not yet been validated or is applicable to your population?

(single choice)

-Yes

-No

Supplementary S2:

State of origin and academic degree of the dietitians surveyed.

Dietitians from each state	Academic degree	n
**Baja California (1.54%, 2)**	Bachelor´s	50% (1)
	Master´s	50% (1)
	Doctorate	0% (0)
**Sonora (0.77%, 1)**	Bachelor´s	0% (0)
	Master´s	0% (0)
	Doctorate	100% (1)
**Chihuahua (2.3%, 3)**	Bachelor´s	33.3% (1)
	Master´s 33.3% (1)	33.3% (1)
	Doctorate 33.3% (1)	33.3% (1)
**Sinaloa (0.77%, 1)**	Bachelor´s	100% (1)

	Master´s	0% (0)
	Doctorate	0% (0)
**Tamaulipas (60.8%, 78)**	Bachelor´s	74.4% (58)
	Master´s	20.5% (16)
	Doctorate	5.1% (4)
**Nuevo Leon (1.54%, 3)**	Bachelor´s	66.6% (2)
	Master´s	33.3% (1)
	Doctorate	0% (0)
**Jalisco (4.6%, 6)**	Bachelor´s	83.3% (5)
	Master´s	16.7% (1)
	Doctorate	0% (0)
**Edo. de Mexico (12.3%, 16)**	Bachelor´s	68.8% (11)
	Master´s	18.8% (3)
	Doctorate	12.5% (2)
**Morelos (0.77%, 1)**	Bachelor´s	0% (0)
	Master´s	100% (1)
	Doctorate	0% (0)
**Puebla (1.54%, 2)**	Bachelor´s	0% (0)
	Master´s	100% (2)
	Doctorate	0% (0)
**Tlaxcala (6.9%, 9)**	Bachelor´s	66.7% (6)
	Master´s	22.2% (2)
	Doctorate	11.1% (1)
**Hidalgo (0.77%, 1)**	Bachelor´s	100% (1)
	Master´s	0% (0)
	Doctorate	0% (0)
**Guanajuato (0.77%, 1)**	Bachelor´s	100% (1)
	Master´s	0% (0)
	Doctorate	0% (0)
**Veracruz (3.1%, 4)**	Bachelor´s	100% (4)
	Master´s	0% (0)
	Doctorate	0% (0)
**Chiapas (0.77%, 1)**	Bachelor´s	100% (1)
	Master´s	0% (0)
	Doctorate	0% (0)
**Yucatán (0.77%, 1)**	Bachelor´s	100% (1)
	Master´s	0% (0)
	Doctorate	0% (0)
